# Celebrating the Third Year of *Small Science*: A Journey of Innovation and Excellence

**DOI:** 10.1002/smsc.202300287

**Published:** 2024-01-28

**Authors:** Small Science Editorial Team

As we step into the fourth year of *Small Science*, it is with immense pride and gratitude that we reflect on the remarkable strides we have made as a journal dedicated to the forefront of scientific discovery. Our commitment to fostering innovation and sharing groundbreaking research remains unwavering, and we are excited to share some significant developments with our esteemed readership.

We bid a heartfelt farewell to Ulf Scheffler, our outgoing Editor in Chief, and editors Sneha K Rhode, Jiaqi Li, and James Cook, whose contributions have been instrumental in shaping the success of *Small Science*. Their dedication to maintaining the journal's high standards and commitment to excellence has set a solid foundation for the future. We wish them the best of luck in their new roles at Wiley. Ulf will continue to support the journal in his new role as Research Integrity Manager and will serve as a consulting Editor for *Small Science*.

Stepping into the role of Editor in Chief from November 2023 is Ekaterina Perets. With her wealth of experience in leading position for sister journal, Small, and for *Advanced Materials*, and vision for the future, we are confident in her ability to lead *Small Science* to new heights. We extend our deepest gratitude to the outgoing team and offer a warm welcome to Ekaterina.

## An Impressive Debut: First Impact Factor Soars to 12.7

We are thrilled to announce that *Small Science* has achieved an impressive first impact factor of 12.7! This remarkable accomplishment is a testament to the high‐quality research published in the journal and the dedication of our authors, reviewers, and editorial team.


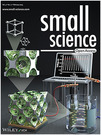
 
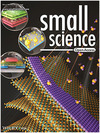
 
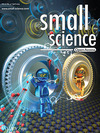


Since its launch, *Small Science* saw the emergence of many highly cited articles, showcasing the impact and relevance of the research published in our journal. Take a look at the top five **Table** **1**. Additionally, we are excited to highlight the top ten articles most accessed manuscripts published in our journal, indicating the broader societal impact and engagement of our research community.

**Table 1:** Highly cited articles in *Small Science*

Title	Authors	Information
High‐Voltage Electrolyte Chemistry for Lithium Batteries	Kanglong Guo, Shihan Qi, Huaping Wang, Junda Huang, Mingguang Wu, Yulu Yang, Xiu Li, Yurong Ren, Jianmin Ma	Full‐text views: 5981 Citations: 34
Protein Engineering and High‐Throughput Screening by Yeast Surface Display: Survey of Current Methods	Joanan Lopez‐Morales, Rosario Vanella, Elizabeth A. Appelt, Sarah Whillock, Alexandra M. Paulk, Eric V. Shusta, Benjamin J. Hackel, Chang C. Liu, Michael A. Nash	Full‐text views: 609 AM score: 3
Electrocatalytic C‐N Coupling for Urea Synthesis	Chen Chen, Nihan He, Shuangyin Wang	Citations: 46
Carbon Dots in Bioimaging, Biosensing and Therapeutics: A Comprehensive Review	Boyang Wang, Huijuan Cai, Geoffrey I. N. Waterhouse, Xiaoli Qu, Bai Yang, Siyu Lu	Full‐text views: 4556 Citations: 53 AM score: 3
Electrohydrodynamic Jet Printing: Introductory Concepts and Considerations	Nhlakanipho Mkhize, Harish Bhaskaran	Citations: 22 AM score: 6
High‐Entropy Nanomaterials for Advanced Electrocatalysis	Sol A Lee, Jeewon Bu, Jiwoo Lee, Ho Won Jang	Full‐text views: 2807 Citations: 5
Branching Ionizable Lipids Can Enhance the Stability, Fusogenicity, and Functional Delivery of mRNA	Kazuki Hashiba, Yusuke Sato, Masamitsu Taguchi, Sachiko Sakamoto, Ayaka Otsu, Yoshiki Maeda, Takuya Shishido, Masao Murakawa, Arimichi Okazaki, Hideyoshi Harashima	Full‐text views: 6129 Citations: 1 AM score: 111

Our commitment to cutting‐edge research continues with the Special Issue on Advances in Thermoelectric Materials Research, which is scheduled to be published in early 2024. This collection will bring together leading experts to explore the latest advancements in this critical field, fostering collaboration and innovation.

*Small Science* proudly presents our special collection “ *Small Science* Editors’ Choice”, which highlights the favourite articles of our esteemed editorial team.

We are humbled and grateful that researchers continue to choose *Small Science* for publishing their cutting‐edge research. This continued commitment is not only reflected in a high number of citations but also by outstanding access and download numbers for our manuscripts.


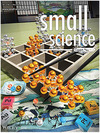
 
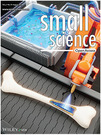
 
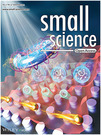


## Planned in 2024: A Glimpse into the Future

Looking ahead, we are excited about upcoming special issues in several countries, showcasing diverse perspectives and advancements in materials science. Our planned special series, including Editor's Choice, will continue to highlight exceptional contributions from researchers across the globe.

To ensure transparency and reproducibility, we have introduced Data Reporting Checklists in our journals for solar cells, batteries, machine learning, and biomedical and life science, setting a benchmark for rigorous and accountable research.

Regarding post‐acceptance, we are implementing changes in workflows and teams, aligning with our vision for continued excellence in the future.

Finally, we invite you to explore the articles in Issue 1, 2024, where cutting‐edge research and groundbreaking discoveries await. *Small Science* remains committed to providing a platform for the exchange of knowledge that shapes the future of science.

As we celebrate the past and look forward to the future, we express our gratitude to our editorial board, authors, reviewers, and readers for their continued support. *Small Science* is not just a journal; it is a community of innovators shaping the landscape of scientific discovery. Here's to another year of pushing boundaries, fostering collaboration, and advancing the frontiers of science.

We wish you all a happy and healthy year 2024!

On behalf of the editorial team,

Ulf Scheffler (Senior Manager Research Integrity & Case Resolution; Consulting Editor)

Ekaterina Perets (Editor‐in‐Chief)

